# Transcriptomic Characterization of an Infection of *Mycobacterium smegmatis* by the Cluster A4 Mycobacteriophage Kampy

**DOI:** 10.1371/journal.pone.0141100

**Published:** 2015-10-29

**Authors:** Andrew Halleran, Samuel Clamons, Margaret Saha

**Affiliations:** Department of Biology, College of William and Mary, Williamsburg, Virginia, United States of America; Niels Bohr Institute, DENMARK

## Abstract

The mycobacteriophages, phages that infect the genus Mycobacterium, display profound genetic diversity and widespread geographical distribution, and possess significant medical and ecological importance. However, most of the majority of functions of mycobacteriophage proteins and the identity of most genetic regulatory elements remain unknown. We characterized the gene expression profile of Kampy, a cluster A4 mycobacteriophage, during infection of its host, *Mycobacterium smegmatis*, using RNA-Seq and mass spectrometry. We show that mycobacteriophage Kampy transcription occurs in roughly two phases, an early phase consisting of genes for metabolism, DNA synthesis, and gene regulation, and a late phase consisting of structural genes and lysis genes. Additionally, we identify the earliest genes transcribed during infection, along with several other possible regulatory units not obvious from inspection of Kampy's genomic structure. The transcriptional profile of Kampy appears similar to that of mycobacteriophage TM4 but unlike that of mycobacteriophage Giles, a result which further expands our understanding of the diversity of mycobacteriophage gene expression programs during infection.

## Introduction

Mycobacteriophages are dsDNA viruses that infect bacteria of the genus Mycobacterium. First isolated from soil samples in 1946 [1], the mycobacteriophages have been studied as a system for detection and diagnosis of tuberculosis [2–4], as a source of molecular tools for engineering Mycobacteria [5–8], and as a model for bacteriophage evolution and genetic diversity [9–14]. Although the mycobacteriophage host with the most clinical relevance is *Mycobacterium tuberculosis*, the most common host used for mycobacteriophage study is *Mycobacterium smegmatis*, a fast-growing model for *M*. *tuberculosis*.

Largely due to science education initiatives such as the SEA-PHAGES and PHIRE programs, an abundance of genomic data exists for the mycobacteriophages. As of the middle of 2015, 5,914 mycobacteriophages have been discovered and 853 sequenced through these initiatives [14]. These phages are grouped into clusters and subclusters determined by overall genomic nucleotide similarity [15]. In order to understand the proteomes of these phages, putative mycobacteriophage proteins are grouped into protein families, known as phams, by shared amino acid similarity [16].

However, despite this wealth of genomic data, the functions of most putative phams remain unknown and even less is known regarding the expression and regulation of bacteriophage genes. Mass-spectrometry and protein interaction studies have been employed to confirm gene calls, identify new gene products, and elucidate gene function [17–20]. Transcriptomic investigations have characterized host responses to infection and identified promoter locations, transcriptional start sites, and small non-coding RNA products [21–26]. Oligonucleotide microarray study of T4 enabled the clustering of genes into distinct temporal stages of infection and the identification of novel promoter sequences [24]. Also utilizing microarray technology, Ainsworth *et al*.’s investigation of Tuc2009 and c2 infection of *L*. *lactis* confirmed previously identified promoter sequences [25]. RNA-Seq analysis of LUZ19 during infection of *P*. *aeruginosa* identified novel transcriptional start sites that confirmed σ^70^ binding site predictions [26].

To our knowledge only one mycobacteriophage, Giles, has been subjected to global transcriptomic analysis with RNA-Seq [27], which allows global gene expression analysis in a manner similar to microarrays but with higher accuracy, especially for low-abundance transcripts, and allows single nucleotide resolution and identification of novel transcripts [28]. Giles belongs to cluster Q, which is a small, tightly conserved cluster (>98% nucleotide similarity between members) and only distantly related to other mycobacteriophage clusters [29]. Indeed, Giles in particular was studied in large part because of its unusual genome architecture and evolutionary dissimilarity from other mycobacteriophages. To better understand the transcriptome-level dynamics of the most common mycobacteriophages, we employed RNA-Seq and mass spectrometry to analyze the infection of *M*. *smegmatis* with a phage with virtually no nucleotide identity with and highly dissimilar genomic organization from Giles.

Kampy is a cluster A phage of the A4 subcluster, discovered in 2012 under the SEA-PHAGES initiative [14]. Cluster A is the most common mycobacteriophage cluster and A4 the second most common subcluster among the cluster A phages, making Kampy highly representative of the mycobacteriophages. Kampy has a 51,378 bp genome with 88 genes and 64% GC content with, like other cluster A phages, a siphoviridae morphotype.

RNA-Seq analysis of an infection of *Mycobacterium smegmatis* by mycobacteriophage Kampy reveals that, as with mycobacteriophage Giles, transcription of the Kampy genome is largely divided into two phases. Transcription of an early-phase suite consisting primarily of genes for nucleotide metabolism and genome replication begins as early as five minutes after infection. A second, late-phase suite consisting of structural genes and lysins is activated around 30 minutes into infection. We observed transcriptional read-through well into suites of genes with opposite orientation and also identify two additional putative transcriptional units.

## Results

### Viral latent period determination

**Fig 1:**
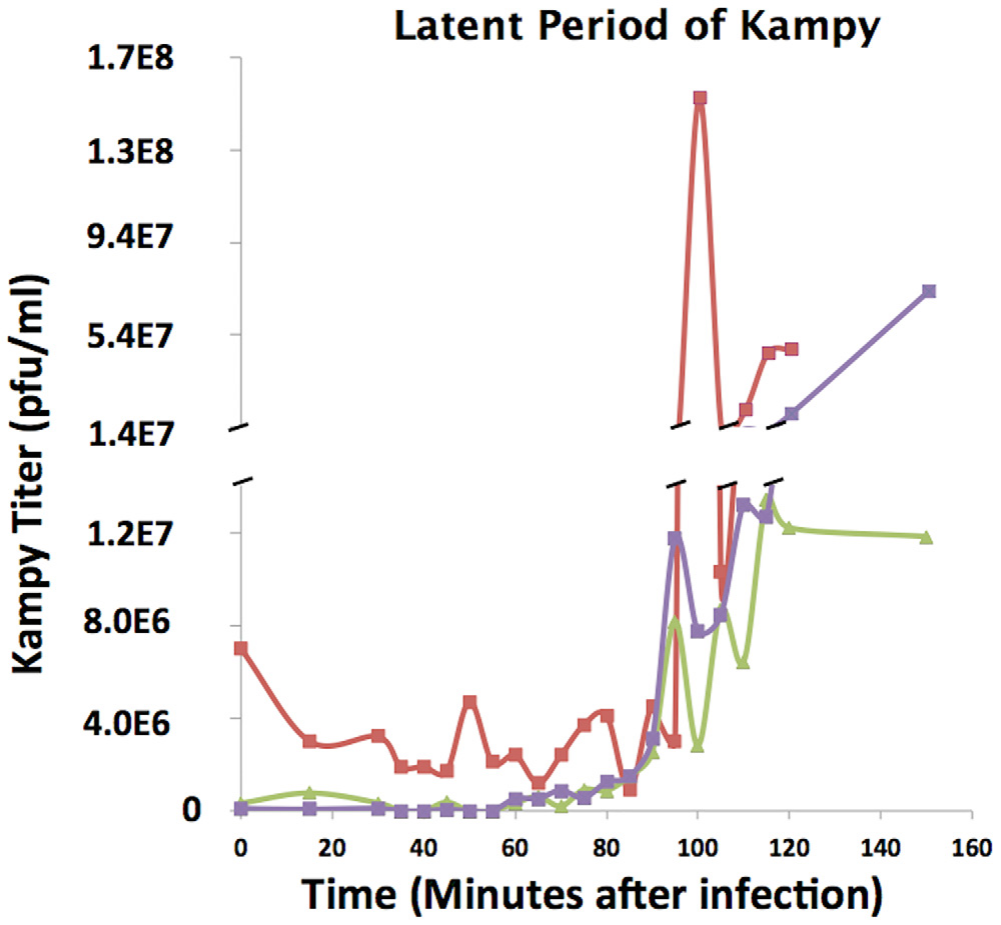
Latent Period Determination. Latent period determination for Kampy. Phage titers were measured at twenty-two time points throughout infection. The three lines represent separate biological replicates.

To determine the latent period of mycobacteriophage Kampy, phage titer in an infected liquid culture of *M*. *smegmatis* was measured at 0, 15, and 30 minutes following infection, then every five minutes until two hours after infection, and then once more at 2.5 hours after infection. Across all three biological replicates, a spike in Kampy titer was observed between 95 and 100 minutes after infection (Fig 1).

### Progression of Viral Transcript Abundance

**Fig 2:**
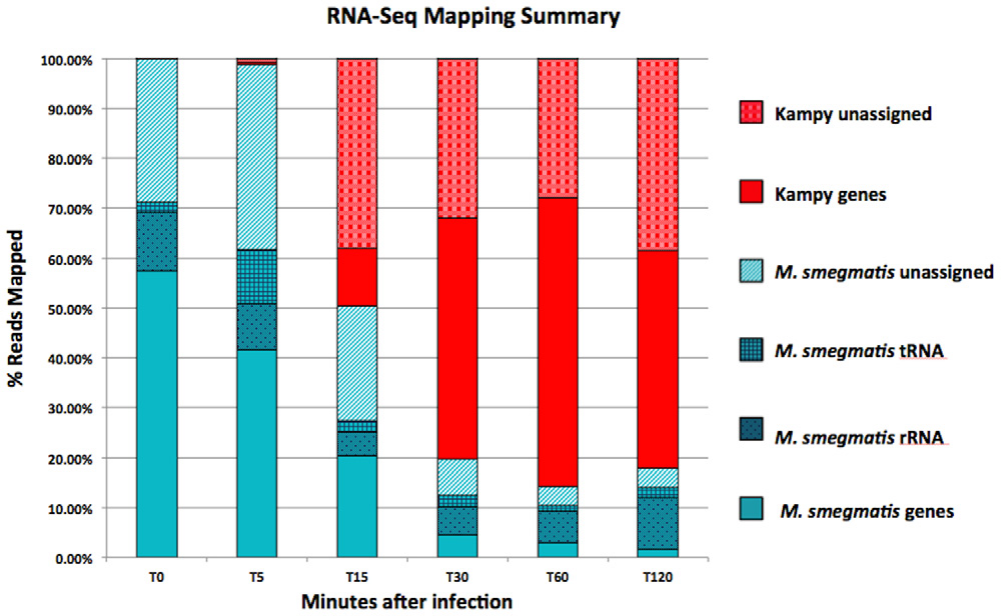
Read Mapping Summary. Summary of percentages of total reads mapped to the *Mycobacterium smegmatis* or Mycobacteriophage Kampy genome before infection and at 5, 15, 30, 60 and 120 minutes post-infection. Biological replicates were averaged for each time point.

RNA-Seq was performed before infection and after addition of virions both before (5, 15, 30, and 60 minutes after infection) and after (120 minutes after infection) predicted lytic burst with two biological replicates at each time point. To prevent reads from *M*. *smegmatis* from incorrectly mapping to regions of Kampy with moderate homology, reads were mapped simultaneously to the *Mycobacterium smegmatis* and mycobacteriophage Kampy genomes. All reads (that successfully mapped) were mapped uniquely. We detected viral mRNA transcripts as early as five minutes after infection, at which point they constituted 1.3% of the total fraction of non-ribosomal reads mapped to either of the Kampy or *Mycobacterium smegmatis* genomes (Fig 2). Fifteen minutes into infection, Kampy mRNA constituted 52% of such mapped non-ribosomal reads. The percentage of viral mRNA increased to approximately 85% of mapped non-ribosomal reads by 30 minutes after infection and stabilized to 92% of mapped non-ribosomal reads from 60 minutes after infection onward.

### Patterns of Viral Gene Expression

**Fig 3:**
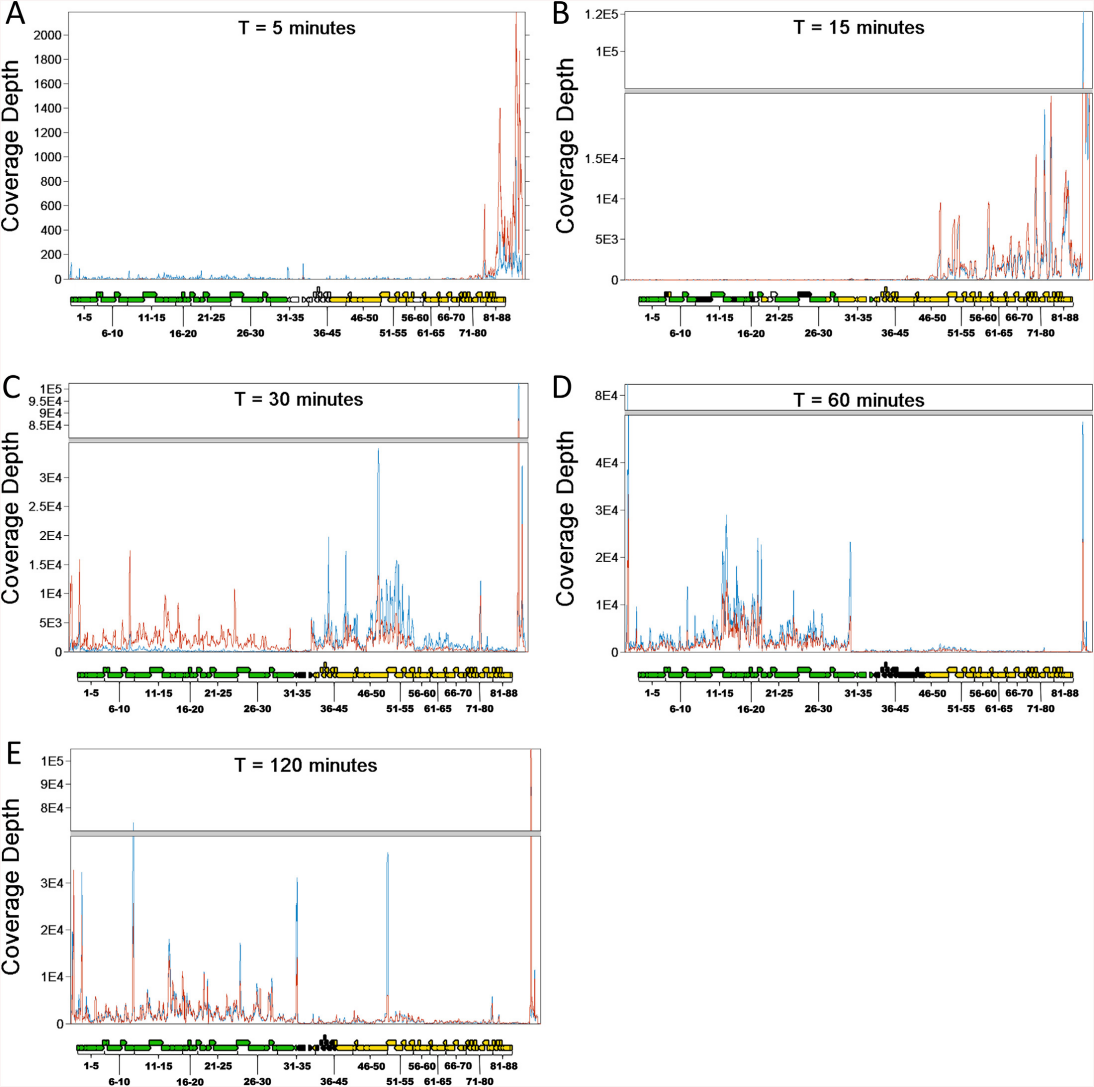
Read Coverage. RNA-Seq coverage of the Kampy genome at 5 (a), 15 (b), 30 (c), 60 (d), and 120 (e) minutes. Profiles are shown for two replicates at each time point. Locations of coding sequences (CDSs) in Kampy are shown below each coverage profile. Coloring of the CDSs indicates transcriptional direction: not transcribed (white), transcribed left to right (green), transcribed right to left (orange) or transcribed in both directions (black). Coloring of the traces (red and blue) represent biological replicates. Coverage is displayed in units of reads per million total non-ribosomal reads mapped to either of the Kampy or *Mycobacterium smegmatis* genomes (RPM), a natural normalized measure of per-base read depth.

At five (Fig 3a) and fifteen minutes (Fig 3b) post-infection, gene expression was observed almost exclusively on the right arm of the genome, which contains genes involved in DNA replication (DNA polymerase, DNA primases, DnaB-like helicases), integration (recombination endonuclease VII), nucleotide metabolism (deoxycytidylate deaminase, thymidylate synthase/thyX, ribonucleotide reductase, phosphoribosyl transferase), host immunity repression, and DNA methylation. Specifically, 86.2% and 99.9% of viral gene expression at five and fifteen minutes post-infection, respectively, occurred upstream of gene 35, as measured by integrating read depth from the beginning of gene 36 to the end of the genome. At both five and fifteen minutes after infection, gene 78, a DNA methylase, is notably (2X-10X) more highly expressed than any other gene with known function.

Five minutes after infection, expression was largely confined to genes 82 through 88, all of which encode hypothetical proteins, and with the highest expression of any gene occurring in gene 83, encoding another hypothetical protein. Fifteen minutes post-infection we observed a leftward shift in expression, indicated by a roughly 3-fold increase in expression of genes 50 through 82 (Table 1). At this time, gene 78 (DNA methylase) was the most highly transcribed gene, and the other DNA methylase (gene 77) was the 13th-most highly transcribed. Other highly expressed genes (Reads Per Kilobase per Million mapped reads (RPKM) > 5,000) at 15 minutes after infection included a metallophosphoesterase (gene 52), a DnaB-like helicase (gene 63), a RecB-like helicase (gene 67), a phosphoribosyl transferase (gene 62), and a ribonucleotide reductase (gene 50).

**Table 1:** Differential expression of genes between times.

	Fold-Difference (P-Value)			
Gene name	T5 to T15	T15 to T30	T30 to T60	T60 to T120
PBI_KAMPY_1_HNH_Endonuclease	-9.4 (0.938)	-229.4 (0.013)	4 (2.14E-04)	-1.1 (0.78)
PBI_KAMPY_2_Hypothetical_Protein	-45.4 (0.972)	-432.3 (2.84E-03)	2.9 (0.019)	1.6 (0.588)
PBI_KAMPY_3_Hypothetical_Protein	∞ (1)	-464 (5.69E-03)	4.3 (1.24E-05)	-1.5 (0.083)
PBI_KAMPY_4_Hypothetical_Protein	-264.1 (0.866)	-2999.3 (0.032)	3.1 (0.111)	-1.1 (0.79)
PBI_KAMPY_5_Hypothetical_Protein	-∞ (0.94)	-∞ (0.016)	5.2 (1.21E-05)	-1.6 (0.039)
PBI_KAMPY_6_Hypothetical_Protein	-∞ (1)	-∞ (0.039)	4.5 (1.68E-03)	-1.3 (0.29)
PBI_KAMPY_7_Hypothetical_Protein	0 (NA)	-∞ (0.093)	5 (5.23E-04)	-1.9 (0.02)
PBI_KAMPY_8_Lysin_A	-178.4 (0.938)	-4659.5 (0.012)	5.2 (2.67E-05)	-2.2 (8.86E-04)
PBI_KAMPY_9_Holin	-∞ (0.866)	-∞ (0.06)	4.6 (1.68E-03)	-1.2 (0.637)
PBI_KAMPY_10_Lysin_B	-26.2 (0.972)	-913.9 (0.042)	3.7 (0.025)	-1.6 (0.053)
PBI_KAMPY_11_Terminase	-37.4 (0.938)	-760.3 (0.042)	4.4 (3.25E-03)	-1.8 (0.015)
PBI_KAMPY_12_Portal	-23.7 (0.972)	-742.1 (0.055)	7.1 (2.86E-06)	-2.6 (3.72E-05)
PBI_KAMPY_13_Capsid_Maturation_Protease	-95.9 (0.866)	-660.7 (0.066)	9.3 (6.22E-12)	-2.6 (2.32E-05)
PBI_KAMPY_14_Scaffolding	-69.3 (0.938)	-623.1 (0.078)	16.4 (1.24E-17)	-4.8 (8.88E-12)
PBI_KAMPY_15_Major_Capsid_Protein	-66.5 (0.94)	-724.3 (0.064)	12.3 (8.53E-15)	-3.8 (8.09E-09)
PBI_KAMPY_16_Hypothetical_Protein	-15.3 (1)	-121.9 (0.136)	11.5 (2.19E-13)	-3.9 (8.09E-09)
PBI_KAMPY_17_Hypothetical_Protein	-122.7 (0.938)	-504.5 (0.087)	21.3 (6.55E-07)	-3.9 (1.98E-04)
PBI_KAMPY_18_Hypothetical_Protein	0 (NA)	-∞ (0.164)	12.3 (3.37E-12)	-1.7 (0.053)
PBI_KAMPY_19_Hypothetical_Protein	∞ (1)	-216.1 (0.117)	15.6 (5.81E-17)	-3.6 (2.58E-08)
PBI_KAMPY_20_Hypothetical_Protein	-∞ (0.921)	-∞ (0.084)	19.7 (1.43E-15)	-4.5 (1.84E-10)
PBI_KAMPY_21_Hypothetical_Protein	-∞ (0.938)	-∞ (0.059)	15.7 (5.81E-17)	-3.7 (1.66E-08)
PBI_KAMPY_22_Major_Tail_Subunit	-245.3 (0.938)	-2166 (0.059)	14.4 (1.83E-16)	-2.8 (7.29E-06)
PBI_KAMPY_23_Tail_Assembly_Chaperone	0 (NA)	-∞ (0.223)	5.1 (2.53E-02)	-2.6 (3.65E-03)
PBI_KAMPY_24_Tail_Assembly_Chaperone	∞ (1)	-492.8 (0.087)	7.1 (2.68E-08)	-1.8 (0.016)
PBI_KAMPY_25_Tapemeasure	-91.2 (0.938)	-821.3 (0.076)	7.6 (4.71E-07)	-2.4 (1.98E-04)
PBI_KAMPY_26_Minor_Tail_Protein	-51.1 (0.972)	-718.2 (0.076)	8.7 (3.05E-06)	-3.1 (1.31E-06)
PBI_KAMPY_27_Minor_Tail_Protein	-75.3 (0.938)	-449.8 (0.089)	13.6 (1.21E-15)	-2.5 (6.88E-05)
PBI_KAMPY_28_Minor_Tail_Protein	-9 (1)	-87.9 (0.132)	8.1 (9.79E-11)	-1.1 (0.714)
PBI_KAMPY_29_Minor_Tail_Protein	-∞ (1)	-∞ (0.109)	15.6 (9.32E-07)	-3.9 (1.40E-05)
PBI_KAMPY_30_Minor_Tail_Protein	-33.4 (1)	-187.6 (0.115)	11.5 (4.85E-08)	-2.2 (7.86E-03)
PBI_KAMPY_31_Minor_Tail_Protein	-44.2 (0.94)	-186 (0.089)	17.8 (1.24E-17)	-2.8 (1.09E-05)
PBI_KAMPY_32_Hypothetical_Protein	∞ (1)	-1.5 (0.558)	7.1 (2.86E-06)	-2 (0.039)
PBI_KAMPY_33_Integrase_28S_int_29	-18.6 (0.972)	-31.5 (3.17E-08)	5.7 (8.41E-08)	-1.7 (0.035)
PBI_KAMPY_34_Hypothetical_Protein	∞ (1)	-5.7 (4.99E-03)	5.4 (1.68E-06)	-2.4 (2.91E-03)
PBI_KAMPY_35_Hypothetical_Protein	∞ (1)	-77.6 (2.19E-13)	-1.5 (0.373)	-1.3 (0.412)
PBI_KAMPY_36_Deoxycytidylate_Deaminase	∞ (1)	-176.3 (1.26E-29)	-3.2 (4.85E-05)	1.5 (0.275)
PBI_KAMPY_37_Hypothetical_Protein	0 (NA)	-∞ (4.32E-08)	1.2 (0.721)	-3 (7.86E-03)
PBI_KAMPY_38_Hypothetical_Protein	∞ (1)	-346.4 (1.54E-19)	-1.5 (0.29)	1.1 (0.828)
PBI_KAMPY_39_Hypothetical_Protein	0 (NA)	-∞ (0.03)	-1.1 (0.924)	-1.5 (0.728)
PBI_KAMPY_40_Hypothetical_Protein	∞ (1)	-295.6 (1.81E-24)	-3.2 (4.17E-05)	1.2 (0.648)
PBI_KAMPY_41_Hypothetical_Protein	∞ (1)	-415.5 (2.69E-32)	-3.2 (4.10E-05)	1.2 (0.637)
PBI_KAMPY_42_Hypothetical_Protein	-1.5 (1)	-94.3 (6.91E-28)	-3.8 (2.86E-06)	1.3 (0.566)
PBI_KAMPY_43_Hypothetical_Protein	-12.1 (1)	-84.1 (3.64E-23)	-4.4 (6.39E-07)	2.8 (0.111)
PBI_KAMPY_44_Hypothetical_Protein	∞ (1)	-121 (3.20E-14)	-2.9 (2.45E-03)	1.3 (0.641)
PBI_KAMPY_45_DNA_Polymerase	1.4 (1)	-32.2 (1.47E-25)	-2.8 (7.78E-05)	1.5 (0.145)
PBI_KAMPY_46_HTH_DNA_Binding_Protein	∞ (1)	-42.2 (2.58E-21)	-3.4 (1.47E-05)	1.7 (0.079)
PBI_KAMPY_47_Hypothetical_Protein	∞ (1)	-15.6 (1.68E-13)	-4 (8.54E-06)	2.1 (0.027)
PBI_KAMPY_48_ThyX	2 (1)	-26.4 (9.43E-21)	-3 (3.26E-05)	-1.1 (0.746)
PBI_KAMPY_49_Hypothetical_Protein	-3.2 (1)	-93.7 (1.40E-05)	-1 (0.864)	-1 (0.888)
PBI_KAMPY_50_Ribonucleotide_Reductase	15.7 (0.036)	-3.7 (5.99E-05)	-2 (7.08E-03)	2.2 (0.582)
PBI_KAMPY_51_Hypothetical_Protein	24.4 (4.13E-03)	-1.8 (0.123)	-2 (9.04E-03)	1.3 (0.291)
PBI_KAMPY_52_Metallophosphoesterase	86.3 (2.52E-09)	-1.5 (0.164)	-3.1 (1.23E-05)	1.5 (0.111)
PBI_KAMPY_53_Hypothetical_Protein	∞ (0.046)	-2.5 (0.006)	-2.1 (9.00E-03)	1.3 (0.436)
PBI_KAMPY_54_DNA_Primase	50.7 (4.78E-03)	-1.4 (0.242)	-1.5 (0.113)	-1.2 (0.669)
PBI_KAMPY_55_DNA_Primase	4 (1)	-3.6 (2.47E-04)	-2.1 (0.016)	1.2 (0.637)
PBI_KAMPY_56_Hypothetical_Protein	2 (1)	-4.7 (4.21E-04)	-3.1 (1.31E-03)	1.8 (0.186)
PBI_KAMPY_57_Endo_VII	∞ (0.938)	-2.4 (0.025)	-1.4 (0.31)	1.3 (0.416)
PBI_KAMPY_58_Hypothetical_Protein	∞ (1)	-1.4 (0.922)	2.1 (0.688)	-1.1 (0.952)
PBI_KAMPY_59_Hydrolase	37.1 (0.044)	1.3 (0.455)	-1.5 (0.146)	1.2 (0.582)
PBI_KAMPY_60_Hypothetical_Protein	183 (1.10E-04)	3.6 (0.003)	-1.8 (0.056)	1.7 (0.055)
PBI_KAMPY_61_Hypothetical_Protein	∞ (0.726)	1 (0.705)	-1.8 (0.31)	1.4 (0.269)
PBI_KAMPY_62_Phosphoribosyl_Transferase	16.8 (0.012)	2.6 (0.045)	-1.3 (0.622)	1.4 (0.321)
PBI_KAMPY_63_DnaB-Like_Helicase	∞ (3.05E-08)	∞ (0.032)	-1.7 (0.087)	1.5 (0.188)
PBI_KAMPY_64_Hypothetical_Protein	∞ (1)	2.1 (0.649)	2.9 (0.512)	-2.4 (0.669)
PBI_KAMPY_65_Hypothetical_Protein	∞ (0.891)	3.1 (0.079)	1.2 (0.717)	1.1 (0.771)
PBI_KAMPY_66_Hypothetical_Protein	∞ (0.938)	-1.4 (0.397)	-1.6 (0.287)	-1.3 (0.46)
PBI_KAMPY_67_RecB-like_helicase	25.9 (2.94E-05)	3.5 (7.57E-05)	-1.2 (0.611)	1.2 (0.529)
PBI_KAMPY_68_Hypothetical_Protein	35.5 (1.70E-04)	5.3 (1.22E-07)	1.2 (0.512)	-1 (0.939)
PBI_KAMPY_69_Immunity_Repressor	∞ (4.13E-03)	2.9 (1.15E-03)	1.2 (0.759)	-1.2 (0.678)
PBI_KAMPY_70_Hypothetical_Protein	∞ (0.972)	2.1 (0.087)	-1.8 (0.372)	2.2 (0.145)
PBI_KAMPY_71_Hypothetical_Protein	∞ (1)	1.6 (0.494)	1 (0.995)	1.7 (0.29)
PBI_KAMPY_72_Hypothetical_Protein	15.4 (0.444)	2.8 (0.087)	-1.5 (0.412)	2.1 (0.016)
PBI_KAMPY_73_Hypothetical_Protein	6 (1)	5 (8.29E-05)	1.1 (0.759)	2.1 (0.09)
PBI_KAMPY_74_Hypothetical_Protein	13 (1)	2.1 (0.079)	1.2 (0.832)	1.3 (0.529)
PBI_KAMPY_75	15.6 (0.972)	2.2 (0.029)	-1.2 (0.866)	2.6 (0.045)
PBI_KAMPY_76_Hypothetical_Protein	5.2 (1)	3.2 (0.01)	-1.3 (0.872)	1.5 (0.582)
PBI_KAMPY_77_DNA_Methylase	7.2 (0.15)	6.5 (2.31E-05)	-2.4 (0.008)	3.2 (5.82E-05)
PBI_KAMPY_78_DNA_Methylase	2.7 (0.866)	17.3 (2.46E-18)	-2.9 (0.017)	6.4 (1.15E-04)
PBI_KAMPY_79_Hypothetical_Protein	1.3 (1)	6.5 (1.07E-06)	-1.1 (0.809)	1.6 (0.312)
PBI_KAMPY_80_SprT-like	-1 (1)	8.4 (4.14E-10)	-1.1 (0.936)	1.7 (0.135)
PBI_KAMPY_81_Hypothetical_Protein	-1.2 (0.972)	1.8 (0.109)	-2.3 (0.158)	6 (3.29E-05)
PBI_KAMPY_82_Hypothetical_Protein	1.5 (1)	20.7 (4.52E-08)	-1.3 (0.611)	1.7 (0.145)
PBI_KAMPY_83_Hypothetical_Protein	-3 (0.345)	13 (1.68E-13)	-1.4 (0.622)	1.5 (0.412)
PBI_KAMPY_84_Hypothetical_Protein	-2 (0.921)	15.9 (2.31E-05)	-1.6 (0.547)	3.1 (0.028)
PBI_KAMPY_85_Hypothetical_Protein	-1.7 (1)	15.8 (0.029)	2.1 (0.547)	1.3 (0.842)
PBI_KAMPY_86_Hypothetical_Protein	-3.2 (0.744)	9 (4.14E-10)	-1.3 (0.688)	1.2 (0.648)
PBI_KAMPY_87_Hypothetical_Protein	-2.5 (0.345)	18.3 (7.32E-18)	1.3 (0.611)	1.1 (0.669)
PBI_KAMPY_88_Hypothetical_Protein	-3 (0.972)	2.4 (0.071)	-1.5 (0.725)	1.9 (0.416)

Differential expression of Kampy from 5 to 15 minutes, 15 to 30 minutes, 30 to 60 minutes, and 60 to 120 minutes. Genes changing from 0 reads to >0 reads are shown with fold-difference ∞. Genes changing from >0 reads to 0 reads are shown with fold-difference -∞. Genes with no expression at either time are shown with fold-difference 0.

Thirty minutes into infection we observed a further shift in transcriptional activity towards the center of the genome (Fig 3c). While Kampy genes 50–88 were most highly expressed at fifteen minutes after infection, at thirty minutes, a region spanning genes 36 through 55 dominated expression. Metallophosphoesterase (gene 52) and ribonucleotide reductase (gene 50) remained very highly expressed (average RPKM 45,027 and 39,010, respectively), but the expression of both DNA methylases decreased relative to that of other genes. We also observed transcriptional activity on the left side of the genome, which primarily contains genes involved in virion construction (terminase, portal protein, capsid maturation protease, scaffolding protein, major capsid protein, major and minor tail subunits, tail assembly chaperones, and tapemeasure protein) and virion escape (two lysins and a holin).

Sixty minutes post-infection, nearly all (~95%) gene expression was observed in the left arm of the genome (Fig 3d). At this time, the highest single peak of expression occurred in a non-coding region upstream of gene 1, although the region upstream of gene 88 remained strongly expressed. Genes 1–31 were all considerably more expressed (18-fold by integrated transcript coverage) than the right-arm genes, with the highest expression spanning genes 14 through 22, as well as a large spike at the 3' end of gene 31 (a minor tail protein). The most highly expressed genes at this stage were a scaffolding protein (gene 14), a major capsid protein (gene 15), and a major tail subunit (gene 22).

**Fig 4:**
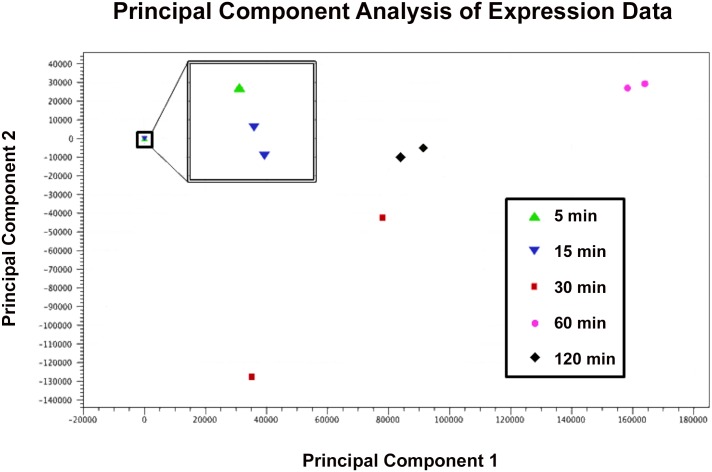
Principal Component Analysis. Principal component analysis on RNA-Seq samples from 5 (green triangle), 15 (blue inverted triangle), 30 (red square), 60 (pink circle), and 120 (black diamond) minutes after infection, showing tight clustering of biological replicates relative to between-time-point variation. Samples 30 minutes post-infection show the most variability, suggesting that transition from early- to late-phase gene expression occurs around this time.

By two hours into infection we observed gene expression on both sides of the genome. Expression was consistent with a superposition of expression patterns at thirty and sixty minutes into infection with the pattern dominated by the latter. This suggests transcription from a second round of viral infections as well as a large number of residual transcripts from the initial infection (Fig 3e). Visualization with principal component analysis (PCA) (Fig 4) suggests that gene expression patterns two hours after infection are intermediate between those at thirty minutes into infection and those at sixty minutes into infection. Notably, the genes with the highest expression from the right-hand arm of the genome are gene 50 (ribonucleotide reductase), gene 51 (hypothetical protein) and gene 52 (metallophosphoesterase), while both DNA methylases were expressed to a lesser degree. This pattern is more similar to expression seen at 30 minutes after infection than at either 5 or 15 minutes, consistent with a latent period of approximately 90 minutes.

Notably, at all time points, including 60 minutes after infection, where expression is largely confined to the left arm of the genome, read coverage was highest in an uncalled region upstream of gene 88 at the far end of the right arm of the Kampy genome (41%, 51%, 16%, 5%, and 14% of transcription as measured by integrated base coverage at 5, 15, 30, 60, and 120 minutes after infection, respectively). Another region at the opposite end of the genome has similarly high read coverage at later stages. These regions display no coding potential according to the bioinformatic tools Glimmer and GeneMark. BLAST comparison shows that the left- and right-hand peaks are conserved within 100% and 96% of sequenced cluster A4 phages, respectively, but are not conserved between other subclusters or clusters. Proteomic analysis revealed no peptide matches to these regions in any translation frame. These data suggest that there are transcribed regions of the genome that are not translated during any stage of viral infection. The high level of expression of these regions could be partially attributable to the presence of a large 5’ read pileup, indicating a transcriptional start site near 50,400 bp and another near 200 bp [30].

Centroid-based clustering with the Xmeans algorithm suggests 16 clusters of gene expression (S1 Table) [32]. No patterns are apparent from Xmeans clustering based on known functional similarity or genomic proximity. Promoter discovery using DNA master (http://cobamide2.bio.pitt.edu/) and BPROM [33] yielded no predicted promoters with scores above 0.6 and 1, respectively, in intergenic regions with the correct orientations. Promoter discovery likely failed because both of these programs use consensus sequences for sigma factor binding sites that are known in *E*. *coli* but not in *M*. *smegmatis*.

### Confirmation of bioinformatically predicted coding regions

To confirm predicted gene annotations in Kampy previously identified only through bioinformatic detection of coding potential, we performed proteomic analysis on samples from 30 minutes into infection, 60 minutes into infection, and 120 minutes into infection. We confirmed the presence of 19 translated open reading frames (ORFs): metallophosphoesterase (gene 52), portal protein (gene 12), major capsid protein (gene 15), major tail subunit (gene 22), scaffolding protein (gene 14), tail assembly chaperone (gene 24) and 12 ORFs corresponding to hypothetical proteins (Table 2). We then compared detected peptides to bioinformatically predicted genes. In all but one case the bioinformatic predictions were verified, with peptides appearing in expected reading frames. Unexpectedly, at 60 minutes after infection (but not 30 or 120 minutes after infection) we detected a protein with several common modifications of the proteins were also detected by the PLGS such as methionine oxidation and carbamidomethylation of the cysteines by the iodoacetamide alkylating agent corresponding to a single continuous ORF spanning the end of gene 66 and the beginning of gene 65, but out of frame with respect to either gene annotation (genomic position 42,331–42,037, reverse orientation). This region was matched with a ProteinLynx Global SERVER (PLGS) score of 1013 and 68% coverage, both scores implying very high confidence in the call. No conserved domains were discovered by comparison of this region to known conserved protein families using HHPred [34]. BLAST searches did not reveal any known proteins with similar sequences. Both genes 65 and 66 show high coding potential according to Glimmer and GeneMark, while the region covered by this frameshifted product does not (in the shifted frame).

**Table 2:** Summary of proteomic analysis of Kampy infection.

	T30		T60		T120	
Gene	PLGS Score^1^	Percent Coverage	PLGS Score	Percent Coverage	PLGS Score	Percent Coverage
Hypothetical Protein (PBI_KAMPY_47)	883	85.23%	N/A	0%	N/A	0%
Hypothetical Protein (PBI_KAMPY_51)	787	86.62%	N/A	0%	N/A	0%
Hypothetical Protein (PBI_KAMPY_56)	N/A	0%	1324	68.63%	N/A	0%
Hypothetical Protein (PBI_KAMPY_65)	N/A	0%	1013	68.37%	N/A	0%
Hypothetical Protein (PBI_KAMPY_53)	N/A	0%	N/A	0%	1493	63.71%
Hypothetical Protein (PBI_KAMPY_3/4)	N/A	0%	N/A	0%	427	49.36%
Hypothetical Protein (PBI_KAMPY_86)	2217	50.62%	1527	63.58%	N/A	0%
Metallophosphoesterase (PBI_KAMPY_52)	2851	87.20%	3071	84.78%	N/A	0%
Hypothetical Protein (PBI_KAMPY_42)	1713	30.64%	N/A	0%	440	39.88%
Hypothetical Protein (PBI_KAMPY_41)	N/A	0%	3029	82.35%	2801	67.23%
Portal (PBI_KAMPY_12)	N/A	0%	901	74.49%	964	68.83%
Hypothetical Protein (PBI_KAMPY_16)	1886	67.81%	7321	68.97%	4355	66.67%
Hypothetical Protein (PBI_KAMPY_41)	1538	93.91%	1611	77.03%	925	68.92%
Hypothetical Protein (PBI_KAMPY_44)	790	61.46%	1188	91.62%	427	34.64%
Hypothetical Protein (PBI_KAMPY_49)	4177	74.87%	5811	71.36%	1687	76.38%
Major Capsid Protein (PBI_KAMPY_15)	4088	68.34%	18676	82.44%	2771	82.76%
Major Tail Subunit (PBI_KAMPY_22)	5705	65.66%	17090	61.62%	17342	62.63%
Scaffolding (PBI_KAMPY_14)	4495	49.74%	18862	62.05%	9911	49.74%
Tail Assembly Chaperone (PBI_KAMPY_24)	3542	76.51%	3431	69.80%	2379	18.79%

^1^PLGS scores were calculated using the ProteinLynx Global Server software.

Summary of mass spectrometry performed on samples during infection at 30 minutes (T30), 60 minutes (T60) and 120 minutes (T120).

Of the proteins we detected, 12 are expressed in the early-phase arm including metallophosphoesterase (gene 52) and 11 genes with unknown function (gene 47, gene 51, gene 56, gene 65/66, gene 53, gene 86, gene 52, gene 42, gene 41, gene 44, gene 49), while 8 are expressed late during infection and involved in virion structure and assembly (gene 12, portal; gene 15, major capsid protein; gene 22, major tail subunit; gene 14, scaffolding; and gene 24, tail assembly chaperone) or have unknown function (gene 3/4, gene 17, gene 16). Structural proteins found at our earliest time point are likely fragments of the virions originally used for infection. The genes associated with the proteins identified via mass spectrometry analysis were all highly expressed according to RNA-Seq. For example, at 30 minutes into infection, of the ten most highly expressed genes based on RNA-Seq, five were detected as proteins. Genes identified as highly expressed in the RNA-Seq experiment were also detected as proteins at similar levels at other time points.

## Discussion

Nearly a thousand mycobacteriophage genomes have been fully sequenced, illuminating the landscape of genetic diversity among these phages. The transcriptional profiles of mycobacteriophage infection, however, remain poorly understood. We performed global gene expression analysis of the cluster A4 mycobacteriophage Kampy’s infection of *Mycobacterium smegmatis*, describing the sequence and approximate timing of transcriptional events during infection.

Viral transcription begins as early as 5 minutes following infection, and by 30 minutes after infection, viral mRNA constitutes the vast majority (>80%) of the transcriptional pool. The dominance of viral transcripts could be caused by highly active viral transcription, degradation of the host’s transcriptome, repression of host transcription, or, more likely, a combination of these mechanisms. As RNA-Seq data are normalized to the total number of mapped reads, they cannot be used to quantify total transcript abundance. Therefore, our data only reveal changes in ratios of expression, not absolute differences between conditions. Experiments that assay RNA degradation and the rate of new transcription will be required to distinguish among the possibilities listed above.

The most closely related phage to mycobacteriophage Kampy that has been subjected to transcriptional analysis is the well-studied cluster A2 mycobacteriophage L5. Lee *et al*. have shown that L5 has two primary arms of transcription driven in part by two strong promoters, one at each end, which drive transcription inwards [35, 36]. While Kampy’s transcriptional pattern is not surprisingly similar to that of L5, comparison of the exact temporal sequence of gene expression between Kampy and L5 is uninformative because L5 is a temperate phage and can form lysogens. The cluster K2 mycobacteriophage TM4, however, is strictly lytic and therefore can be more appropriately compared to Kampy. Similar to both L5 and Kampy, the TM4 genome is separated into a left arm, containing genes primarily involved in virion assembly, and a right arm, containing genes involved in DNA replication and metabolism. Unlike Kampy and L5, every gene in TM4 is transcribed entirely left to right throughout the entire genome [37]. Ford *et al*. observe translation of early-phase proteins between 10 and 20 minutes after infection, followed by the production of late-phase proteins sometime between 30 and 60 minutes after infection. The transcriptional program of Kampy appears to follow a similar progression, with activation of the early-phase genes 5 minutes following infection and the late-phase gene expression onset between 30 and 60 minutes after infection.

While mycobacteriophages Giles and Kampy share a common bacteriophage genome architecture consisting of a contiguous set of early-phase genes and a contiguous set of late-phase genes, transcription of these sets is strikingly different [29]. Transcription in Giles is largely unidirectional, with 82% of genes transcribed left to right at all time points measured [27]. In contrast, we observed changes over time in Kampy’s directional transcriptional patterning. Genes 1–24 are exclusively transcribed in a left to right manner and genes 49–88 are similarly transcribed exclusively right to left, regardless of the time point. However, the region in the middle of the genome (genes 25–48) is transcribed in both directions in proportions that vary with respect to the progression of infection.

Although a two-promoter model could be used to explain much of Kampy’s transcriptional profile, clustering analysis suggests that this is insufficient to completely explain observed dynamics and additional transcriptional units remain to be identified. Indeed, the presence of higher levels of coverage at 30 and 60 minutes after infection at genes 13–23 and 48–56 respectively suggest putative transcriptional units for further investigation.

While we observed agreement between biological replicates at most times, at thirty minutes into infection, expression varied considerably between replicates. Specifically, early-phase transcripts from the right hand arm dominated expression in one replicate, whereas in the other replicate we observed transcription across the entire genome. These data suggest that a shift in gene expression occurs around thirty minutes into infection, which would be consistent with the expression timing of TM4 [37]. The variability between replicates may also suggest variability in the exact timing of expression. Higher temporal resolution sampling would quantify biological variability in infection progression from early- to late-phase gene expression.

Transcription beyond thirty minutes was dominated by late-phase transcription from the left arm of the genome. However, we still observed low levels of early-phase transcription from the right arm at these times, a finding consistent with those of Dedrick *et al*. for mycobacteriophage Giles and of Lavigne *et al*. in an infection of *Pseudomonas aeruginosa* by LUZ19 [27, 28]. As previously noted for virus-host transcript ratio, our data only reveal differences in ratios of expression, not absolute differences. It is therefore unclear whether early phase gene expression remains constant but much lower than subsequent late phase expression or, alternatively, whether early phase genes are actively repressed or degraded later in infection.

Notably, for each side of the genome, when that side of the genome is transcribed, we observed abundant transcription of a few hundred bases on that particular side of the genome upstream of any region with coding potential. With the exception of one sample at 120 minutes, these peaks consistently contained the highest levels of transcription on each side of the genome. Read pileups at transcriptional start sites are known to occur in RNA-Seq due to overrepresentation of 5’ ends after RNA fragmentation [30]. We propose that the high coverage observed on either end of the Kampy genome are mostly likely the result of transcriptional start sites for two promoters.

In addition to transcriptomic analysis, we provide protein-level confirmation of the presence of eighteen previously annotated genes. Twelve of these confirmed proteins have no known function, as well as no known homology outside the mycobacteriophages. This work provides the first experimental confirmation of the translation of these twelve genes from twelve distinct protein phams with a total of 1837 members, whose existence was previously only predicted through bioinformatic detection of coding potential. Our proteomic analysis also identified a protein product spanning parts of genes 66 and 65 that was out-of-frame with respect to both genes. This product is encoded immediately downstream of a site similar to a translational frameshift site previously reported in mycobacteriophage TM4 (CCGAAAA and GGGAAAA, respectively) [31]. Further study will be required to determine whether this product is truly functional.

Using RNA-Seq and mass spectrometry, we have broadly described the transcriptional program of a cluster A4 mycobacteriophage, Kampy. Our analysis shows much greater similarity between the infection dynamics and timing of Kampy and the K2 cluster mycobacteriophage TM4 than between Kampy and the cluster Q mycobacteriophage Giles. Our high-throughput sequencing approach suggests functional studies that can further clarify mechanisms of mycobacteriophage gene regulation during infection.

## Materials and Methods

### Bacterial strains and infection conditions

All experiments were performed using *Mycobacterium smegmatis* strain mc^2^155 (ATCC 700084). Cells were grown in Middlebrook 7H9 media with AD supplement (10%), carbenicillin (50 μg/ml), cyclohexamide (10 μg/ml), and calcium chloride (1 mM).

For all infections, log-phase liquid-culture *M*. *smegmatis* was incubated with high-titer lysate of mycobacteriophage Kampy. For latent period experiments, Kampy was added at 1:10 multiplicity of infection (MOI) to ensure complete adsorption of viral particles, thus reducing background virions that would be detected during the assay. For RNA-Seq and proteomic experiments, Kampy was added at a 10:1 MOI to maximize the fraction of infected hosts. Infected cells were grown in a shaking incubator at 37°C.

### Latent Period Experiment

*M*. *smegmatis* log-phase liquid-culture was combined with high-titer lysate of mycobacteriophage Kampy at 1:10 MOI. Infections were incubated for 5 minutes at 37°C in a shaking incubator to allow adsorption of viral particles, and then pelleted by centrifugation for 3 minutes at 5,000G at 4°C. Cells were washed three times with 1 ml room temperature phage buffer (10 mM Tris pH 7.5, 10 mM MgSO4, 4% w/v NaCl) to remove unadsorbed phage particles, centrifuging at 5,000 G for 3 minutes at 4°C between each wash. Pellets were resuspended in 40 ml Middlebrook 7H9 media supplemented as described above. Infected cells were incubated at 37°C in a shaking incubator for the duration of the experiment. At each measured time point, 200 μl of infected culture was serially diluted in phage buffer, added to 500 μL of uninfected *M*. *smegmatis*, and incubated at room temperature for 5 minutes before plating for measurement of viral titer.

### RNA preparation

For each time point, 15 ml of infected *M*. *smegmatis* culture was pelleted by centrifugation for 10 minutes at 400 G at 4°C. Pellets were flash frozen in liquid nitrogen and stored at -80°C until further processing. Frozen cell pellets were resuspended in 1 ml TRIzol (Life Technologies). Cells were then disrupted as previously described [27]. Total RNA was extracted from the TRIzol solution with bromochloropropane, washed with isopropanol, precipitated with 70% ethanol, and resuspended in nuclease-free water.

To prepare samples for RNA-Seq, total RNA was incubated with recombinant DNase I (Ambion) and RNasin (Promega) for 30 minutes at 37°C. DNase I was inactivated by addition of EDTA (5 mM) and 10 minute incubation at 70°C.

### RNA-Seq

Ribosomal RNA was depleted from prepared samples by subtractive hybridization using the RiboZero kit for gram-positive bacteria (Illumina) and depleted RNA was checked for integrity and rRNA depletion with the Bioanalyzer 2100 (Agilent). Libraries for sequencing were prepared from rRNA-depleted RNA using the Ion Total RNA-Seq Kit v2 (Ion Torrent) and sequenced on an Ion Torrent PGM according to the manufacturer's instructions. A total of 4,926,133 reads were generated across all conditions.

Reads with intact adapters were trimmed of adapters using the Torrent Server browser. Trimmed reads were mapped simultaneously to the *M*. *smegmatis* (NCBI NC_018289.1) and mycobacteriophage Kampy (GenBank KJ510414.1) genomes using the “RNA-Seq analysis” tool in CLC Genomic Workbench 7.0.x. To test for differential expression of genes between timepoints, count tables of reads mapping to Kampy genes were exported and rRNA and tRNA entries were removed. These count tables were analyzed using the DESeq package in Bioconductor R [38, 39]. P-values were corrected for multiple hypothesis testing using Benjamini-Hochberg correction with α = 0.05 [40].

Cluster analysis was performed with the Xmeans algorithm implemented in the standalone program kmeans [32]. Analysis was performed with 88 maximum centers and otherwise recommended default arguments.

### Mass Spectrometry

Whole protein was extracted from infected *M*. *smegmatis* culture as previously described [41]. Samples were first brought to pH of 8.0 with 1M ammonium bicarbonate. Samples were then incubated with 10 mM final concentration of tris(2-carboxyethyl)phosphine (TCEP) (Sigma-Aldrich) for 10 minutes at 95°C followed by alkylation in the dark with 15 mM iodoacetamide (IAA) (Sigma-Aldrich) for one hour. Samples were then digested with sequencing grade trypsin (Promega) at a 1:50 enzyme to substrate ratio overnight at 37°C. After overnight digestion, samples were then brought up to a final pH of 2.0 using trifluoroacetic acid (TFA), flash frozen, and then lyophilized until further use.

Prior to mass spec analysis, lyophilized samples were brought up in 1 mL of aqueous buffer containing 2% acetonitrile and 0.1% TFA. Samples were then applied to a C18 TopTip (Glygen) according to manufacturers instructions. Briefly, each C18 TopTip was first equilibrated with 100% acetonitrile followed by washing with buffer containing 2% acetonitrile and 0.1% TFA. The trypsin digested samples were then applied to the TopTip followed by subsequent washing with 2% acetonitrile and 0.1% TFA. Samples were eluted from the C18 TopTip using an aqueous solution containing 60% acetonitrile with 0.1% TFA and were then subjected to concentration to approximately 10μl volume using a speed vac.

Mass spec analysis was performed on a nanoACQUITY Xevo G2 QToF equipped with a TRIZAIC UPLC source (Waters Corp). Samples were loaded onto an Acquity HSS T3 Trizaic nanoTile with dimensions of 85 μm x 100 mm. Samples were analyzed using label-free data-independent acquisition mass spectrometry with subsequent data analyzed using ProteinLynx Global Server V2.3 with a minimum of 3 and 7 ion matches per peptide and protein, respectively. The data was searched against a six-frame translation of the mycobacteriophage Kampy genome.

## Supporting Information

S1 Table Clustering of Kampy gene expression by xmeans. (XLSX)
